# Impact of Age and Sex on CD4+ Cell Count Trajectories following Treatment Initiation: An Analysis of the Tanzanian HIV Treatment Database

**DOI:** 10.1371/journal.pone.0164148

**Published:** 2016-10-07

**Authors:** Arianna R. Means, Kathryn A. Risher, Eva L. Ujeneza, Innocent Maposa, Joseph Nondi, Steven E. Bellan

**Affiliations:** 1 Department of Global Health, University of Washington, Seattle, Washington, United States of America; 2 Department of Epidemiology, Johns Hopkins Bloomberg School of Public Health, Baltimore, Maryland, United States of America; 3 The South African Department of Science and Technology, National Research Foundation Centre of Excellence in Epidemiological Modelling and Analysis (SACEMA), University of Stellenbosch, Stellenbosch, South Africa; 4 Department of Mathematics and Statistics, School of Health and Applied Sciences, Polytechnic of Namibia, Windhoek, Namibia; 5 National AIDS Control Program, Ministry of Health and Social Welfare (MOHSW), Dar es Salaam, The United Republic of Tanzania; 6 Department of Epidemiology and Biostatistics, College of Public Health, University of Georgia, Athens, Georgia, United States of America; University of Medicine and Dentistry of New Jersey - New Jersey Medical School, UNITED STATES

## Abstract

**Objective:**

New guidelines recommend that all HIV-infected individuals initiate antiretroviral treatment (ART) immediately following diagnosis. This study describes how immune reconstitution varies by gender and age to help identify poorly reconstituting subgroups and inform targeted testing initiatives.

**Design:**

Longitudinal data from the outpatient monitoring system of the National AIDS Control Program in Tanzania.

**Methods:**

An asymptotic nonlinear mixed effects model was fit to post-treatment CD4+ cell count trajectories, allowing for fixed effects of age and sex, and an age by sex interaction.

**Results:**

Across 220,544 clinic visits from 32,069 HIV-infected patients, age- and sex-specific average CD4+ cell count at ART initiation ranged from 83–136 cells/mm^3^, long term asymptotic CD4+ cell count ranged from 301–389 cells/mm^3^, and time to half of maximal CD4+ reconstitution ranged from 3.57–5.68 months. CD4+ cell count at ART initiation and asymptotic CD4+ cell count were 1.28 (95% CI: 1.18–1.40) and 1.25 (95% CI: 1.20–1.31) times higher, respectively, for females compared to males in the youngest age group (19–29 years). Older patients started treatment at higher CD4+ counts but experienced slower CD4+ recovery than younger adults. Treatment initiation at greater CD4+ cell counts was correlated with greater asymptotic CD4+ cell counts within all sex and age groups.

**Conclusion:**

Older adults should initiate care early in disease progression because total immune reconstitution potential and rate of reconstitution appears to decrease with age. Targeted HIV testing and care linkage remains crucial for patient populations who tend to initiate treatment at lower CD4+ cell counts, including males and younger adults.

## Introduction

In 2013, 24.7 million (23.5–26.1 million) people were estimated to be living with HIV in sub-Saharan Africa and 37% of those living with HIV were receiving antiretroviral therapy (ART) [1]. In Tanzania, over 1.5 million people are estimated to be infected with HIV, 41% of whom were estimated to have initiated treatment as of 2013 [1, 2]. It is important for HIV-infected individuals to initiate ART in order to improve immune function, slow disease progression [3], decrease risk of HIV transmission [4], and reduce risk of AIDS-associated mortality [5]. These and other benefits associated with ART are in part realized through reductions in HIV viral load and increases in CD4+ T white blood cell counts.

CD4+ cell count has historically been used to guide clinical decision making regarding ART initiation, regimen switching, and clinical monitoring [6]. The most recent 2015 World Health Organization (WHO) treatment guidelines recommend treatment for all HIV-infected individuals regardless of CD4+ cell count [7]. During the period spanning the data analyzed here (2003–2012), Tanzanian treatment guidelines recommended treatment initiation at CD4+ cell counts below 200 cells/mm^3^, below CD4+ cell counts of 350 cells/mm^3^ for individuals in WHO Stage 3, and for all individuals meeting WHO Stage 4 clinical criteria regardless of CD4+ cell count [6].

HIV utilizes and destroys CD4+ cells during viral replication and thus CD4+ cell counts are one clinical indicator of immune function or reconstitution following treatment initiation. Reductions in CD4+ cells and the resulting inflammation and immunosuppression are associated with onset of multiple co-morbid opportunistic infections such as tuberculosis, candidiasis, pneumonia, Kaposi’s sarcoma, and other conditions [8, 9]. However, different opportunistic infections occur at varying levels of CD4+ depletion and immune function [10]. Thus, it is important to understand how CD4+ levels change after treatment initiation in order to understand the changing clinical vulnerabilities of a patient throughout immune reconstitution.

Baseline CD4+ cell counts tend to vary by country; a cross-sectional study in Zimbabwe and Uganda found that among both HIV-uninfected and HIV-infected women, CD4+ cell counts were lower in Zimbabwe than Uganda, after controlling for age, contraceptive method, education, and partner HIV status [11]. However, CD4+ levels also vary within populations and, importantly, CD4+ cell reconstitution following ART initiation may differ by age and sex [10, 12–14].

Once an individual begins treatment CD4+ cell counts generally increase at a constant rate before plateauing [15]. Initial rapid increases in CD4+ cell levels following ART initiation are likely associated with the redistribution of cells stored in the lymphoreticular system [16]. The less rapid increase in cells that follows (i.e. plateauing) is due in part to the generation of naïve CD4+ cells through cell division or from the thymus [17].

Due to the combination of within-individual variability (such as diurnal variation) and measurement error variability (i.e. imperfect instruments), capturing the trends in CD4+ cell count trajectory is challenging. While a number of studies have simply presented descriptive statistics (i.e. mean, median) at time points following ART initiation[18–20], this fails to take into account the longitudinal nature of CD4+ cell count trajectories. Several modeling strategies have been utilized to more rigorously describe CD4+ cell count recovery trajectories following ART initiation. Some have used linear mixed effects models[21–24], which have the drawback of enforcing a linear trend on every individual’s CD4+ cell count trajectory. Additional linear models include spline terms to account for changing slope over time[15, 25], including hierarchical Bayesian change-point models[26]. Some models incorporate smoothing terms [27, 28] or latent trajectory fitting[24], which incorporates additional flexibility but simultaneously limits interpretability of the model terms. Other studies have used three parameter asymptotic nonlinear mixed effects models, which allow for nonlinear CD4+ cell count trajectories [29–31].

Due to the influence of CD4+ cell counts on long term patient outcomes, it is important to understand the impact of baseline CD4+ cell counts and other patient characteristics on long-term immune recovery. Leveraging a Tanzanian national HIV patient data base, this study describes the correlation between patient-level baseline and long-term immune CD4+ cell counts and to identify demographic factors mediating this correlation.

## Methods

### Study design

We analyze longitudinal clinical data from the outpatient monitoring system of the National AIDS Control Program (NACP) in Tanzania. The system utilizes sets of recording and reporting tools and is distinctively centralized, with a national database that recognizes each patient by their unique Care and Treatment Clinic (CTC) ID number from all over the country. All individuals enrolled in care are supplied with client held CTC-1 cards. Elementary records of patient encounters are captured within facility-held CTC-2 cards, which are the foundation of both the paper and electronic systems currently in use.

### Study participants

Our study population includes treatment and morbidity characteristics of HIV-infected adults receiving treatment between 2003 and 2012 at CTCs in three provinces: Mwanza, Dar es Salam and Tanga. We restricted the analysis to individuals 19 years of age or older at ART initiation who had complete patient records that included: patient sex, ART initiation date, age at ART initiation, baseline CD4+ cell count (within 2 months prior or 3 months post-treatment initiation), and five or more documented visits at which CD4+ cell count was measured (excluding visits more than 2 months prior to ART initiation). The data were de-identified and anonymized prior to data analysis. The Ministry of Health and Social Welfare routinely collects this de-identified, anonymous surveillance data. We received approval to conduct these analyses from the Tanzanian National AIDS Control Programme, and a waiver for ethical review from the Johns Hopkins Bloomberg School of Public Health.

### Statistical analysis

We used a hierarchical nonlinear mixed effects (NLME) model to fit CD4+ cell count progression following ART initiation. The model’s fixed effects describe the population average response while the random effects describe patients’ deviation from the population average within a given fixed effect category (i.e. between subject variability).

**Fig 1:**
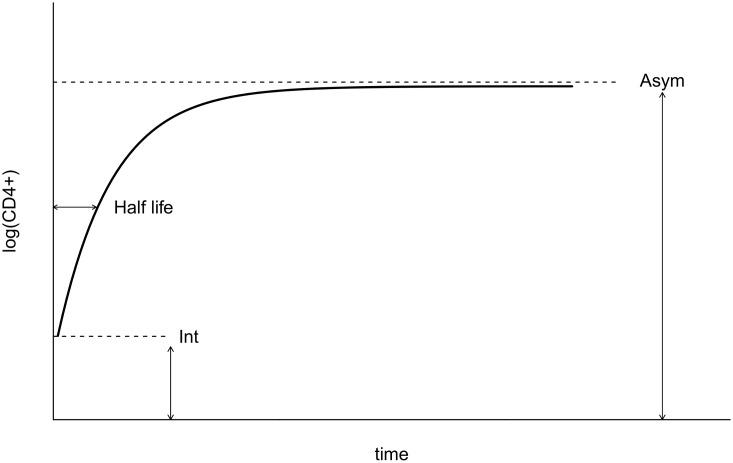
Asymptotic model diagram. Schematic diagram of the negative exponential asymptotic model of CD4+ cell recovery. Int, Asym and the half life (*t*_50%_) represent the CD4+ cell count at initiation of antiretroviral treatment, the asymptotic CD4+ cell count and the time to reach 50% of the maximal CD4+ recovery, respectively.

We used a negative exponential asymptotic function as the basis of the NLME model (Fig 1). Consistent with previous studies of CD4+ cell trajectories, this approach assumes asymptotic behavior for long-term CD4+ cell recovery [29–31]. A recent study of CD4+ cell recovery in pediatric patients found that fits of this asymptotic model did not converge for all patients and, consequently, assumed that lack of convergence indicated that the asymptotic model was an inadequate representation of those patient trajectories. These authors chose to fit linear mixed effects models to those patients instead [32]. However, we expand from this work by noting that lack of convergence when fitting a model indicates only uncertainty that the fitting algorithm identified the best estimates of the model parameters; it does not indicate that the model is an inadequate fit. Lack of convergence is more likely to occur when fitting more complicated models to data that are noisy or sparse (e.g. linear models are simple enough to always converge given at least two observations whereas asymptotic models may not easily converge for small numbers of observations particularly when they are noisy around the asymptotic trend).

We explored these alternatives by simulation. We took the best-fit parameters from our best asymptotic nonlinear mixed model and used that model to simulate patient data. Because these data were simulated from a truly asymptotic model, with observation-level noise and inter-patient variation representative of the real data, they provide a rigorous test case to identify whether lack of convergence of an asymptotic model indeed indicates a poor model or whether instead that lack of convergence could be a symptom of fitting a complicated model to noisy and sparse data. Indeed, we found that the asymptotic model did not converge for 38.5% of simulated (and truly asymptotic) patient trajectories. In contrast, only 18% of data from real patients failed to converge. We thus conclude that failed convergence is not sufficient cause to reject an underlying asymptotic model for patients within our data set and, consequently, analyzed all patients using an asymptotic CD4+ cell recovery model. We further highlight that asymptotic models are fairly flexible three parameter models that can yield approximately linear trajectories such that dividing patients into those for whom it’s easier to fit a linear model versus an asymptotic model is unnecessary.

The asymptotic function used is such that given individual *j*, the logarithm of CD4+ cell count at time *t*_*ij*_ is modelled as

$$\text{log}(CD4_{ij})=\;Asym_{j}-\left(Asym_{j}-\;Int_{j}\right)\text{exp}(-\;c_{j}t_{ij})+\;\varepsilon_{ij}$$ (1)

where *Asym*_*j*_, *Int*_*j*_ and *c*_*j*_ are patient *j* specific parameters, *t*_*ij*_ is the time of follow up in years and *ε*_*i*,*j*_ is the error term.

The parameters in the NLME model represent individual-level biological processes: the intercept *Int*_*j*_ is the logarithm of the CD4+ cell count at ART initiation for patient j, the asymptote *Asym*_*j*_ is the logarithm of the long-term reconstituted CD4+ cell count, and *c*_*j*_ is the rate of exponential decay from the initial log CD4+ cell count upwards to the asymptotic log CD4+ cell count (decay is upwards in the negative exponential asymptotic model). This parameter can be more intuitively understood by considering the half-life, which is the time needed to reach 50% of the total CD4+ cells increase, *t*_50%_ = −ln(0.5)/*c*, or to reach 90% of the total increase, *t*_90%_ = −ln(0.1)/*c*. The error term *ε*_*i*,*j*_ is assumed to be normally distributed and represents variation due to CD4+ cell count measurement error or due to a patient’s day-to-day variation around their average CD4+ cell count trajectory. In order to simplify the results, estimated parameters were exponentiated so that they could be interpreted on a CD4+ cell (and not log(CD4+)) scale.

In our hierarchical asymptotic model, we used Akaike information criterion (AIC) model selection to determine whether to include the effects of sex, age categories, or a sex by age interaction on each of the three asymptotic model parameters (asymptotic CD4+ cell count, CD4+ cell count at ART initiation, and the log rate of CD4+ cell increase), yielding four possible combinations (intercept only, sex only, age only, sex by age interaction) for each asymptotic model parameter. We then considered all 64 possible combinations (4^3^) of these fixed effects for the three asymptotic model parameters. In all models, we also specified that each of the three asymptotic model parameters have a patient-level random effect, with these random effects’ correlations estimated from the data (e.g. patients with lower initial CD4+ cell counts may have lower final CD4+ cell counts, on average, than the average patient within their age-sex strata). A single model was clearly distinguishable as the best model (no other models were within 2 ΔAIC of the best model), and we only present results for this single model. This model included age-by-sex interactions for initial and asymptotic CD4+ count, but only an effect of age on the rate parameter:

$$Int_{j}=\beta_{j,\text{sex},\text{age}}^{Int}+\varepsilon_{j,Int}$$ (2)

$$Asym_{j}=\beta_{j,\text{sex},\text{age}}^{Asym}+\varepsilon_{j,Asym}$$ (3)

$$\text{log}\left(c_{j}\right)=\beta_{j,\text{age}}^{Asym}+\varepsilon_{j,c}$$ (4)

where $\beta_{j,\text{sex},\text{age}}^{Int}$, for example, denotes that effect of being in individual *j*’s age and sex category on the initial log CD4+ count and *ε*_*j*,*Int*_ indicates that individual’s initial log CD4+ count’s random deviation from other individuals in the same age and sex group, with the three random effect terms being multivariate normally distributed. Confidence intervals for all parameters of interest were constructed based on linear combinations of fitted coefficients using their variance covariance matrix and the corresponding variance transformations. All analysis was performed in the statistical environment R (available at: http://www.r-project.org) using the nlme package for nonlinear modeling [33].

## Results

The patient sample analyzed included 220,544 clinic visits from 32,069 HIV-infected patients (Table A in S1 File shows patient exclusion criteria). At baseline, the largest proportion of patients were aged 30–39 while the smallest proportion of patients occurred in the >60 age group. The median CD4+ cell count was 135 (IQR 64–209) and nearly half of patients were classified as WHO Stage 3. Older patients were more likely to initiate treatment at higher CD4+ cell counts and later WHO stages than younger age groups. Over the duration of visits (ranging from October 2003 to September 2012), the mean number of patient visits was 6.9 and patients were followed for a median of 3.7 (IQR 2.8–4.7) years (Table 1 and Table B in S1 File).

**Table 1:** Demographic characteristics and follow-up data of analyzed patients.

Age group	19–29	30–39	40–49	50–59	>60	all
**Number of patients**	5155 (100%)	13927 (100%)	9038 (100%)	3152 (100%)	797 (100%)	32069 (100%)
**Number of visits**	35222	96139	62454	21504	5225	220544
**Average visits/patient**	6.8	6.9	6.9	6.8	6.6	6.9
Median follow-up time per patient (years)	3.6	3.7	3.7	3.7	3.5	3.7
**Number of female patients**	4487 (87%)	10344 (74.3%)	5601 (62%)	1732 (54.9%)	410 (51.4%)	22574 (70.4%)
**Number pregnant (% amongst female patients)**	307 (6.84%)	366 (3.54%)	28 (0.5%)	3 (0.17%)	2 (0.49%)	706 (3.13%)
**Baseline CD4+ count (% within age group)**						
**0–50**	1189 (23.1%)	2964 (21.3%)	1706 (18.9%)	522 (16.6%)	114 (14.3%)	6495 (20.3%)
**51–200**	2512 (48.7%)	7336 (52.7%)	4894 (54.1%)	1700 (53.9%)	434 (54.5%)	16876 (52.6%)
**201–350**	1093 (21.2%)	2789 (20%)	1865 (20.6%)	720 (22.8%)	183 (23%)	6650 (20.7%)
**351–500**	208 (4.03%)	503 (3.61%)	367 (4.06%)	139 (4.41%)	45 (5.65%)	1262 (3.94%)
**500+**	153 (2.97%)	335 (2.41%)	206 (2.28%)	71 (2.25%)	21 (2.63%)	786 (2.45%)
**WHO stage (% within age group)**						
**1**	644 (12.5%)	1480 (10.6%)	749 (8.29%)	286 (9.07%)	59 (7.4%)	3218 (10%)
**2**	1026 (19.9%)	2727 (19.6%)	1722 (19.1%)	621 (19.7%)	142 (17.8%)	6238 (19.5%)
**3**	1914 (37.1%)	5393 (38.7%)	3642 (40.3%)	1268 (40.2%)	331 (41.5%)	12548 (39.1%)
**4**	559 (10.8%)	1617 (11.6%)	1116 (12.3%)	368 (11.7%)	108 (13.6%)	3768 (11.7%)
**Unknown**	1012 (19.6%)	2710 (19.5%)	1809 (20%)	609 (19.3%)	157 (19.7%)	6297 (19.6%)

Nearly three quarters (70.4%) of patients were female. Male patients were most common in the lowest CD4+ strata (0–50 CD4+ cells/mm^3^) at 34.0% and oldest age groups (61+) at 48.6%. Male patients were least common in the highest CD4+ strata (>500 CD4+ cells/mm^3^) at 22.9% and youngest age groups (19–30) at 13% (Table C in S1 File). Very few (3.3%) females were pregnant at ART initiation (Table 1).

Using AIC, our best fit model included an age by sex interaction term for the initial and asymptotic log CD4+ cell counts, and age for the CD4+ cell rate of increase parameter (Table D in S1 File).

### CD4+ cell counts at ART initiation by age and gender

**Fig 2:**
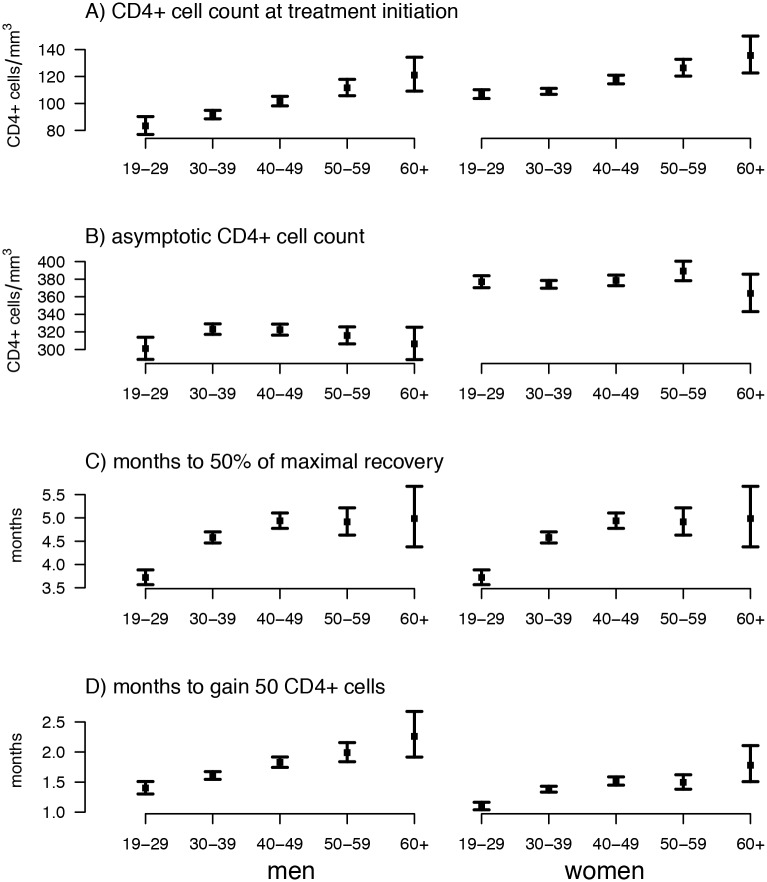
Estimated effects of age and sex on CD4+ cell count response to treatment. Points reflect fixed effects parameters estimated from a hierarchical asymptotic nonlinear mixed effects model with error bars indicating 95% confidence intervals. Numerical values given in Table C in S1 File.

The estimated average CD4+ cell count at ART initiation was 83.4 cells/mm^3^ among 19–29 year old males and increased steadily with age to 121 cells/mm^3^ for >60 year old men, equating to a 1.45-fold (1.26–1.66) differences between these age groups (Fig 2, Tables E and F in S1 File). Within the youngest age group, CD4+ cell count at ART initiation was 107 cells/mm^3^, i.e. 1.28 (95% CI: 1.18–1.40) times higher for females compared to men. CD4+ cell count at ART initiation amongst female patients also increased steadily with age, though this trend was less pronounced, with >60 year old females exhibiting an average of 136 cells/mm^3^.

### Asymptotic CD4+ cell counts by age and gender

The estimated average asymptotic CD4+ cell count (reflecting long-term CD4+ cell recovery) for males aged 19–29 was 301 CD4+ cells/mm^3^. Females in the 19–29 year age group had a 1.25 times greater asymptotic CD4+ cell count relative to males of the same age at 377 cells/mm^3^. Asymptotic CD4+ cell count only varied slightly by age group with 30–49 year old males exhibiting the greatest value amongst males at 323 cells/mm^3^, and 51–60 year old females exhibiting the greatest amongst females at 389 cells/mm^3^.

### Rate of CD4+ cell recovery by age

The rate of CD4+ cell recovery as measured by time until 50% of maximal CD4+ recovery reached was similar between sexes but slower with increasing age with an estimated 3.72 months and 4.99 months until 50% recovery for 19–29 year olds and >60 year old individuals, respectively. When measured as time until a 50 cells/mm^3^ increase since treatment initiation (which accounts for the different amount of CD4+ cells gained across age-sex groups), females recovered more quickly than males and older ages recovered more slowly in both sexes, with 19–29 year old males and females taking 1.4 and 1.1 months, respectively, and >60 males and females taking 2.26 and 1.78 months, respectively, to gain 50 cells/mm^3^.

### Individual variability within age-sex groups

**Fig 3:**
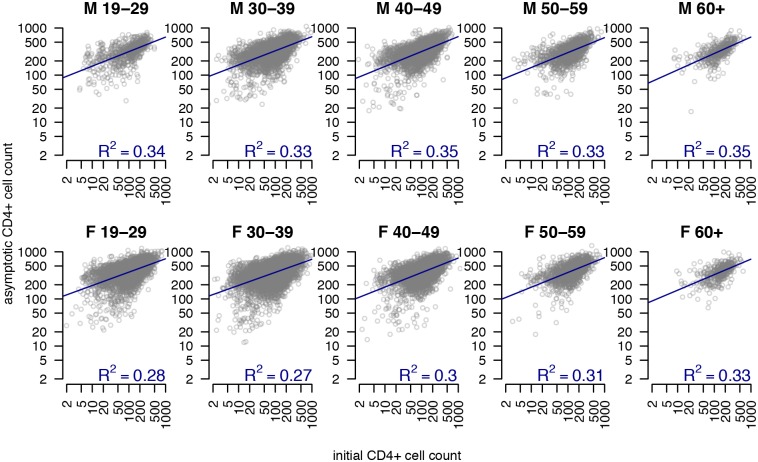
Initial CD4+ cell count and asymptotic CD4+ cell count by age and sex. For each sex and age grouping, these panels show estimated asymptotic CD4+ count versus estimated CD4+ count at treatment initiation, as fitted in the best fit asymptotic non-linear mixed effects regression model of CD4+ counts over time for patients within the outpatient monitoring system of the National AIDS Control Program in Tanzania. Each circle represents one patient. Blue lines show fitted linear regressions with value in bottom right of each panel showing the R^2^ for this correlation.

The standard deviation of clinic observation-level residuals (e.g. *ε*_*ij*_) was 0.58. As characterized by the fitted patient-level random effects, we observed substantial variation between patients in their log CD4+ cell counts at ART initiation (standard deviation, SD: 0.90), their asymptotic log CD4+ cell counts (SD: 0.51), and their log rate of CD4+ cell increase (SD: 0.74). We additionally observed a strong positive correlation between patient-level random effects for initial and asymptotic CD4+ cell counts (correlation on log CD4+ scale: 0.40, 95% CI: 0.39–0.42), such that individuals with a greater CD4+ cell count at ART initiation exhibited a greater long term CD4+ cell count. Notably, this correlation remained consistent when examining patient-level correlations within age-sex groups (Fig 3). We observed a strong positive correlation between amount of CD4+ cells gained (i.e. difference between initial and asymptotic values) and the time taken to recover as measured by the time taken to attain 50% of the maximal CD4+ cells gained (Figure A in S1 File; correlation between *Asym* and log(*c*) estimated as -0.60, 95% CI: -0.62 to -0.58). Finally, there was no notable correlation between the CD4+ cell count at ART initiation and the number of CD4+ cells gained (Figure B in S1 File).

## Discussion

New WHO guidelines recommend that all HIV-infected individuals should initiate ART following an HIV diagnosis [7]. However, clinical decisions to initiate or change treatment may continue to be made at an individual level for the immediate future due to a dearth of medication in low-resource settings and in accordance with country-specific guidelines. This analysis identifies demographic factors underlying patient-level variation in CD4+ cell count trajectories, using an asymptotic nonlinear mixed model, which not only takes into account the asymptotic behavior of long term CD4+ cell counts but also quantifies the between subject variability in the initial and asymptotic CD4+ cell counts as well as the rate of recovery.

In this study, females tended to have higher CD4+ cell counts at ART initiation. This finding is consistent with previous research, including Malaza et al., a recent population-based study in Kwazulu-Natal, South Africa, which found that HIV-negative females had higher CD4+ cell counts than males [34]. Additionally, females in this study tended to achieve a greater CD4+ reconstitution than males (on average between 51–76 cells/mm^3^ higher). Another study from South Africa found that females experienced greater CD4+ cell count increases than males over the 36 months following ART initiation [12]. Evidence also suggests that males experience greater virological failure and mortality rates relative to females after starting treatment, likely due to delayed treatment initiation [35].

We also found that CD4+ cell count at ART initiation increased strongly with patient age at ART initiation for both males and women. This contrasts with Malaza et al.’s population-based study, which found that CD4+ cell counts increased slightly with age in HIV-uninfected individuals but indicated no consistent age pattern in HIV-infected individuals [34]. Thus, it appears that in Tanzania, older individuals may seek care or, once in care, be initiated on ART earlier in their disease course than younger individuals. This may also be partly explained by older individuals progressing to later disease stages at greater CD4+ cell counts and, consequently, meeting treatment threshold criteria at greater CD4+ cell counts than younger individuals. However, despite starting treatment at greater CD4+ levels older age groups did not reach a higher CD4+ recovery asymptote relative to younger age groups. Additionally, older individuals recovered more slowly than younger individuals whether measured as the time taken to attain 50% of their maximal recovery or as time taken to increase 50 CD4+ cells/mm^3^ above their baseline level at treatment initiation. Thus, while older individuals initiate ART at greater CD4+ counts, they appear to recover CD4+ cells at a slower rate and ultimately reach a CD4+ cell count that is no higher than younger individuals. This is may be due to more rapid disease progression [36, 37] and a decrease in the adaptive immune system activity of older adults [38, 39], which may decrease the speed of CD4+ cell recovery.

We observed a positive correlation between patient-level CD4+ cell counts at ART initiation and asymptotic CD4+ cell counts even after accounting for age and sex effects, indicating that individuals who initiate treatment earlier in their disease course (before CD4+ cell count has dramatically decreased) ultimately achieve a higher CD4+ cell count. We did not find any correlation between patient-level CD4+ cell counts at ART initiation and the level of CD4+ cells/mm^3^ gained, indicating that the benefits of starting early are due primarily to lower levels of CD4+ depletion and not greater levels of CD4+ cells gained. Similarly, a study from the United States found that after six years of ART, only patients with CD4+ cell counts of at least 350 cells/mm^3^ at baseline returned to near normal CD4+ levels [40]. These findings were recently corroborated in cohorts in the United States and in Brazil; the weighted CD4+ cell counts of 3116 patients increased for four years following treatment before leveling off, however only 50% of the population attained CD4+ cell counts of at least 449 cells/mm^3^. The individuals with pre-treatment CD4+ cell counts of at least 350 cells/ mm^3^ exhibited increasing CD4+ cell counts over time with 76% of these patients exceeding the CD4+ cell count threshold of more than 500 cells/mm^3^ ten years following ART initiation [18]. This finding is also consistent with findings from a recent study of pediatric HIV patients which associated lower pre-ART CD4+ cell counts with lower long-term CD4+ cell counts [29]. Importantly, we found that the correlation between initial and asymptotic CD4+ count remained consistent across age-sex groups, implying that early ART initiation is beneficial for all sexes and ages.

This study has several limitations. First, selection bias could result from the individuals excluded from our analytic dataset (those with less than 5 clinic visits) being substantially different than the individuals included. Additionally, due to incomplete patient records we were not able to adjust for patient-level ART adherence or regimen.

Our findings suggest that across patients, earlier ART initiation is associated with greater long term CD4+ cell counts. The importance of older adults initiating care early in their disease progression is emphasized by the finding that their immune reconstitution rates are not as high as younger patients. However older patients who start treatment earlier have improved CD4+ trajectories relative to older patients who initiate treatment later. This study also highlights the importance of targeted HIV testing and linkage to care for patient populations who appear to initiate treatment at lower CD4+ cell counts, including males and younger adults. Because younger adults likely contribute substantively to sexual transmission of HIV it is important for these populations to initiate therapy in order to reduce viral load and thus population-level HIV transmission. Younger populations also experience rapid recovery once initiated on therapy, which is important for long-term household livelihood, wellbeing, and community development.

## Supporting Information

S1 File Supplementary Appendix. (DOCX)
