# Correction: Impact of Age and Sex on CD4+ Cell Count Trajectories following Treatment Initiation: An Analysis of the Tanzanian HIV Treatment Database

**DOI:** 10.1371/journal.pone.0170155

**Published:** 2017-01-06

**Authors:** Arianna R. Means, Kathryn A. Risher, Eva L. Ujeneza, Innocent Maposa, Joseph Nondi, Steven E. Bellan

The following information is missing from the Funding section: Partial funding also through Model of Infectious Disease Agent Study National Institution of General Medical Sciences U54GM111274.
