# Supplementary material for: Impact of Age and Sex on CD4+ Cell Count Trajectories following Treatment Initiation: An Analysis of the Tanzanian HIV Treatment Database
Source: PLoS One. 2016 Oct 7;11(10):e0164148. doi: 10.1371/journal.pone.0164148 (PMC5055355; doi:10.1371/journal.pone.0164148)
Supplement: S1 File — (DOCX) [file pone.0164148.s001.docx]

**S1 File**

**S1 Table: Exclusion criteria**

|  | **Percent of patients** | **Number of patients** |
| --- | --- | --- |
| **Total number of observations** | 100 | 593584 |
| **Non-missing CD4** | 66.93 | 397288 |
| **Non-missing sex** | 99.98 | 593488 |
| **Non-missing ART start date** | 56.63 | 336147 |
| **Only one ART start date** | 54.81 | 325351 |
| **Non-missing age at ART start** | 56.58 | 335841 |
| **Non-missing baseline CD4** | 36.88 | 218934 |
| **Over 19 years of age ever in dataset** | 91.11 | 540829 |
| **Over 19 years of age at ART initiation** | 51.43 | 305264 |
| **Meets all above criteria** | 33.57 | 199274 |
| **Meets above criteria and ≥ 5 CD4 observations including and after baseline CD4** | 5.4 | 32,069 |

**S2 Table. Criteria met for ART initiation by age group.**

| age at treatment initiation (years) | 19-29 | 30-39 | 40-49 | 50-59 | >60 | all ages |
| --- | --- | --- | --- | --- | --- | --- |
| only met CD4+ criteria of <200 cells/mm^3^ | 1213 (24%) | 3231 (23%) | 1910 (21%) | 698 (22%) | 139 (17%) | 7191 (22%) |
| only met WHO stage criteria of ≥ stage 3 | 718 (14%) | 1951 (14%) | 1417 (16%) | 527 (17%) | 138 (17%) | 4751 (15%) |
| met both above criteria | 1755 (34%) | 5059 (36%) | 3341 (37%) | 1109 (35%) | 301 (38%) | 11565 (36%) |
| met neither above criteria | 457 (8.9%) | 976  (7%) | 561 (6.2%) | 209 (6.6%) | 62 (7.8%) | 2265 (7.1%) |
| unknown WHO stage | 1012 (20%) | 2710 (19%) | 1809 (20%) | 609 (19%) | 157 (20%) | 6297 (20%) |
| total | 5155 (100%) | 13927 (100%) | 9038 (100%) | 3152 (100%) | 797 (100%) | 32069 (100%) |

**S3 Table. Sex by baseline CD4+ cell count and by age group.**

| Baseline CD4+ cell count | 0-50 | 51-200 | 201-350 | 351-500 | 500+ |
| --- | --- | --- | --- | --- | --- |
| Male | 2207 (34%) | 4960 (29.4%) | 1792 (26.9%) | 356 (28.2%) | 180 (22.9%) |
| Female | 4288 (66%) | 11916 (70.6%) | 4858 (73.1%) | 906 (71.8%) | 606 (77.1%) |
| Total | 6495 (100%) | 16876 (100%) | 6650 (100%) | 1262 (100%) | 786 (100%) |
|  |  |  |  |  |  |
| Age (years) | **19-29** | **30-39** | **40-49** | **50-59** | **>60** |
| Male | 668 (13%) | 3583 (25.7%) | 3437 (38%) | 1420 (45.1%) | 387 (48.6%) |
| Female | 4487 (87%) | 10344 (74.3%) | 5601 (62%) | 1732 (54.9%) | 410 (51.4%) |
| Total | 5155 (100%) | 13927 (100%) | 9038 (100%) | 3152 (100%) | 797 (100%) |

**S4 Table. AIC model selection**

|  | Sex | | | Age | | |  |  | Sex | | | Age | | |  |
| --- | --- | --- | --- | --- | --- | --- | --- | --- | --- | --- | --- | --- | --- | --- | --- |
| model | Asym | Int | c | Asym | Int | c | ΔAIC | **model** | Asym | Int | c | Asym | Int | c | ΔAIC |
| 1 | + | + |  | + | + | + | 0.0 | **33** |  | + | + | + | + | + | 822.0 |
| 2 | + | + |  |  | + | + | 5.5 | **34** |  | + | + |  | + | + | 827.7 |
| 3 | + | + | + | + | + | + | 101.5 | **35** |  | + |  | + | + | + | 850.1 |
| 4 | + | + | + |  | + | + | 122.9 | **36** |  | + |  |  | + | + | 856.6 |
| 5 | + | + |  | + |  | + | 126.0 | **37** |  |  | + | + |  | + | 857.8 |
| 6 | + |  |  |  | + | + | 186.2 | **38** |  | + | + | + | + |  | 893.6 |
| 7 | + |  |  | + | + | + | 190.3 | **39** |  | + | + | + |  | + | 919.7 |
| 8 | + | + | + | + |  | + | 231.0 | **40** |  |  | + | + | + |  | 935.8 |
| 9 | + | + | + |  |  | + | 244.2 | **41** |  | + |  | + | + |  | 935.8 |
| 10 | + |  |  | + |  | + | 266.3 | **42** |  | + |  | + |  | + | 945.9 |
| 11 | + |  |  |  |  | + | 267.6 | **43** |  | + | + |  |  | + | 957.7 |
| 12 | + | + | + | + | + |  | 276.7 | **44** |  | + | + |  | + |  | 964.6 |
| 13 | + |  | + | + | + | + | 291.9 | **45** |  |  |  | + |  | + | 967.6 |
| 14 | + |  | + |  | + | + | 307.3 | **46** |  |  | + |  |  | + | 970.1 |
| 15 | + |  | + | + |  | + | 368.3 | **47** |  | + | + | + |  |  | 977.0 |
| 16 | + | + | + | + |  |  | 375.8 | **48** |  |  |  | + | + |  | 977.3 |
| 17 | + | + |  |  |  | + | 378.1 | **49** |  | + |  |  |  | + | 986.2 |
| 18 | + |  | + |  |  | + | 381.1 | **50** |  |  | + | + |  |  | 999.9 |
| 19 | + | + | + |  |  |  | 434.5 | **51** |  |  |  |  |  | + | 1002.6 |
| 20 | + | + |  | + |  |  | 439.8 | **52** |  | + |  | + |  |  | 1013.7 |
| 21 | + |  | + | + | + |  | 462.2 | **53** |  |  | + |  | + |  | 1014.2 |
| 22 | + |  | + |  | + |  | 489.1 | **54** |  | + |  |  | + |  | 1020.6 |
| 23 | + |  |  | + | + |  | 503.3 | **55** |  |  |  | + |  |  | 1031.3 |
| 24 | + |  | + | + |  |  | 517.1 | **56** |  |  |  |  | + |  | 1057.4 |
| 25 | + |  |  |  | + |  | 520.1 | **57** |  |  |  |  |  |  | 1063.1 |
| 26 | + |  |  | + |  |  | 563.1 | **58** |  | + | + |  |  |  | 1100.2 |
| 27 | + |  | + |  |  |  | 569.2 | **59** |  |  | + |  |  |  | 1125.6 |
| 28 | + |  |  |  |  |  | 603.4 | **60** |  | + |  |  |  |  | 1158.8 |
| 29 |  |  | + | + | + | + | 775.3 | **61** | + | + |  |  |  |  | 57010.8 |
| 30 |  |  | + |  | + | + | 786.7 | **62** | + | + |  |  | + |  | * |
| 31 |  |  |  |  | + | + | 802.2 | **63** | + | + | + |  | + |  | * |
| 32 |  |  |  | + | + | + | 804.8 | **64** | + | + |  | + | + |  | * |

*****AIC not calculable for these three models because we were unable to fit these models (the fitting algorithm would not converge). However, based on AIC values of similar models, these were unlikely to compete for the best model.

AIC model selection table showing model 1 as the clear best model. Given the size of our data set, if including both age group and sex, we always included their interaction (i.e. we did not consider models with both age and sex main effects but no interaction).

**S5 Table. Estimated effects of age and sex on CD4+ cell count response to treatment.**

| Age at treatment initiation (years) | CD4+ count at treatment initiation | | Asymptotic CD4+ cell count | | Exponential decay rate towards asymptotic CD4+ count | |
| --- | --- | --- | --- | --- | --- | --- |
|  | Male | Female | Male | Female | Male | Female |
| 19-29 | 83.4 (77, 90.3) | 107 (104, 110) | 301 (289, 314) | 377 (370, 384) | 2.23 (2.14, 2.33) | 2.23 (2.14, 2.33) |
| 30-39 | 91.7 (88.6, 94.9) | 109 (107, 111) | 323 (317, 329) | 374 (370, 379) | 1.82 (1.77, 1.86) | 1.82 (1.77, 1.86) |
| 40-49 | 102 (98.2, 105) | 118 (115, 121) | 323 (316, 329) | 379 (373, 385) | 1.68 (1.63, 1.74) | 1.68 (1.63, 1.74) |
| 50-59 | 112 (106, 118) | 126 (120, 133) | 316 (306, 326) | 389 (378, 400) | 1.69 (1.59, 1.8) | 1.69 (1.59, 1.8) |
| >60 | 121 (109, 134) | 136 (123, 150) | 306 (289, 325) | 364 (343, 386) | 1.67 (1.46, 1.9) | 1.67 (1.46, 1.9) |
|  |  |  |  |  |  |  |
|  | **Months to 50% of maximal recovery** | | **Months to 90% of maximal recovery** | | **CD4+ cells/mm3 gained** | |
|  | Male | Female | Male | Female | Male | Female |
| 19-29 | 3.72 (3.88, 3.57) | 3.72 (3.88, 3.57) | 12.4 (12.9, 11.8) | 12.4 (12.9, 11.8) | 218 (212, 224) | 270 (267, 274) |
| 30-39 | 4.58 (4.7, 4.46) | 4.58 (4.7, 4.46) | 15.2 (15.6, 14.8) | 15.2 (15.6, 14.8) | 231 (229, 234) | 265 (263, 267) |
| 40-49 | 4.94 (5.11, 4.78) | 4.94 (5.11, 4.78) | 16.4 (17, 15.9) | 16.4 (17, 15.9) | 221 (218, 224) | 261 (258, 264) |
| 50-59 | 4.92 (5.22, 4.63) | 4.92 (5.22, 4.63) | 16.3 (17.3, 15.4) | 16.3 (17.3, 15.4) | 204 (201, 208) | 263 (258, 268) |
| >60 | 4.99 (5.68, 0.38) | 4.99 (5.68, 4.38) | 16.6 (18.9, 14.5) | 16.6 (18.9, 14.5) | 185 (179, 191) | 228 (220, 236) |
|  |  |  |  |  |  |  |
|  | **Months to gain 50 CD4+ cells/mm^3^** | | |  |  |  |
|  | Male | Female |  |  |  |  |
| 19-29 | 1.4 (1.51, 1.3) | 1.1 (1.16, 1.04) |  |  |  |  |
| 30-39 | 1.61 (1.67, 1.55) | 1.38 (1.43, 1.33) |  |  |  |  |
| 40-49 | 1.83 (1.92, 1.74) | 1.52 (1.59, 1.45) |  |  |  |  |
| 50-59 | 1.99 (2.16, 1.84) | 1.5 (1.62, 1.38) |  |  |  |  |
| >60 | 2.26 (2.68, 1.92) | 1.78 (2.11, 1.51) |  |  |  |  |

Values reflect fixed effects parameters estimated from a hierarchical asymptotic nonlinear mixed effects model (95% confidence intervals).

**S6 Table. Relative value of estimated fixed effects parameters to the value of the reference contrast (19-30 year old men) and 95% confidence interval on this relative value.**

| age at treatment initiation (years) | CD4+ count at treatment initiation | | asymptotic CD4+ cell count | | exponential decay rate towards asymptotic CD4+ count | |
| --- | --- | --- | --- | --- | --- | --- |
|  | male | female | male | female | male | female |
| 19-29 | 1 (1, 1) | 1.28 (1.18, 1.4) | 1 (1, 1) | 1.25 (1.2, 1.31) | 1 (1, 1) | 1 (1, 1) |
| 30-39 | 1.1 (1.01, 1.2) | 1.31 (1.2, 1.42) | 1.07 (1.03, 1.12) | 1.24 (1.19, 1.3) | 0.813 (0.773, 0.854) | 0.813 (0.773, 0.854) |
| 40-49 | 1.22 (1.12, 1.33) | 1.41 (1.3, 1.54) | 1.07 (1.02, 1.12) | 1.26 (1.2, 1.31) | 0.754 (0.714, 0.796) | 0.754 (0.714, 0.796) |
| 50-59 | 1.34 (1.22, 1.47) | 1.52 (1.38, 1.67) | 1.05 (0.996, 1.11) | 1.29 (1.23, 1.36) | 0.757 (0.704, 0.815) | 0.757 (0.704, 0.815) |
| >60 | 1.45 (1.27, 1.66) | 1.63 (1.43, 1.85) | 1.02 (0.946, 1.1) | 1.21 (1.12, 1.3) | 0.746 (0.651, 0.856) | 0.746 (0.651, 0.856) |

**S1 Fig**. **Months to 50% of maximal recovery versus** **CD4+ cells gained versus**


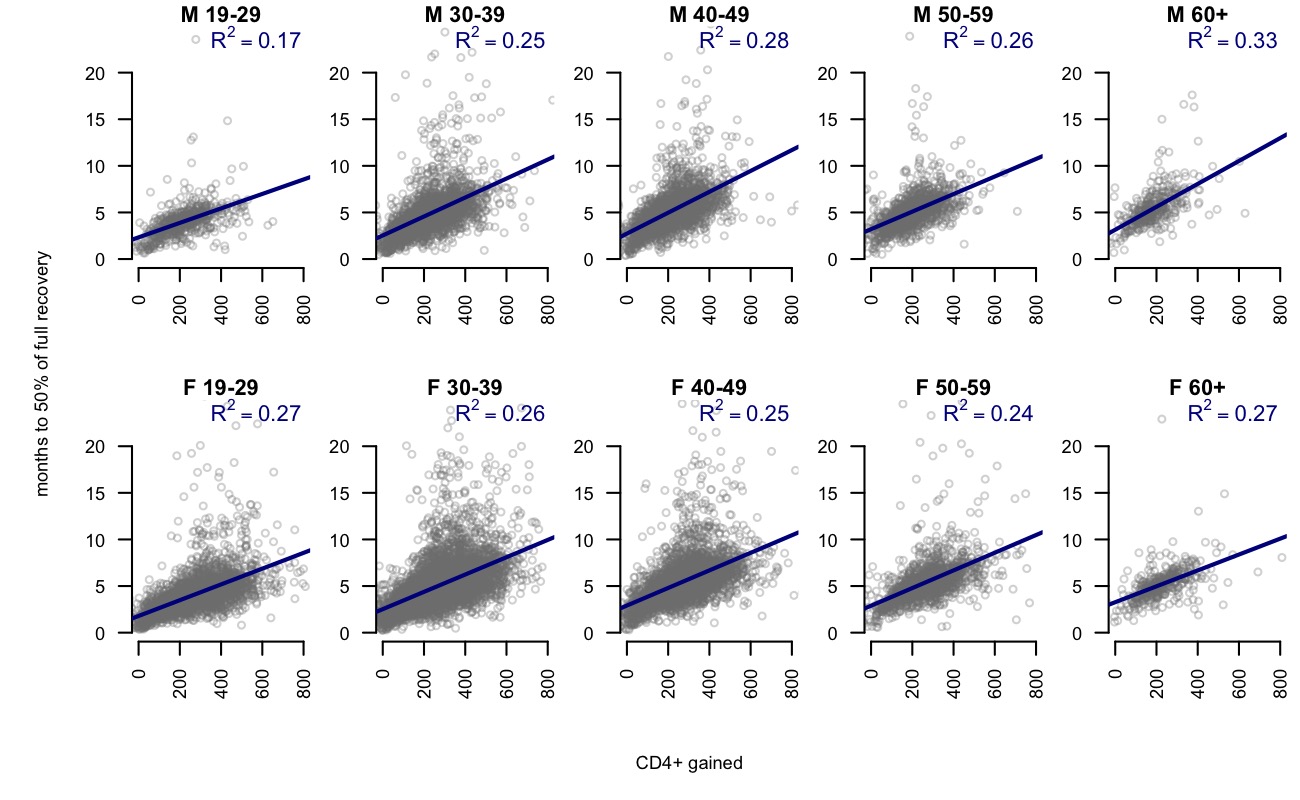


For each sex and age grouping, these panels show estimated CD4+ count at treatment initiation vs. estimated maximal CD4+ cells/mm^3^ gained (i.e. at a patient’s asymptote), as fitted in the best fit asymptotic non-linear mixed effects regression model of CD4+ counts over time for patients within the outpatient monitoring system of the National AIDS Control Program in Tanzania. Each circle represents one patient. Blue value in bottom right of each panel showing the R^2^ for the correlation between these values, indicating that patients that gain more CD4+ cells take longer to reach this greater recovery.

**S2 Fig**. **CD4+ cells gained versus initial CD4+ count**


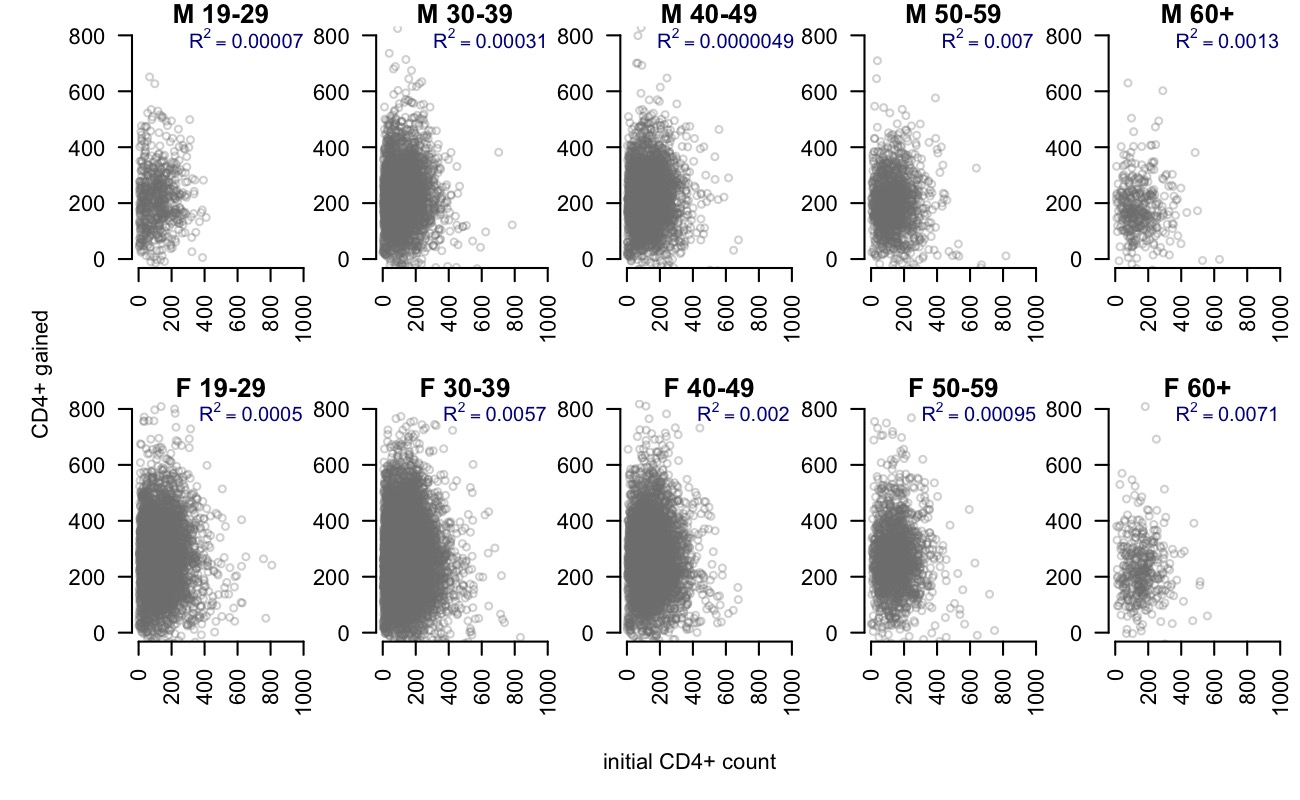


For each sex and age grouping, these panels show estimated CD4+ count at treatment initiation vs. estimated maximal CD4+ cells/mm^3^ gained (i.e. at a patient’s asymptote), as fitted in the best fit asymptotic non-linear mixed effects regression model of CD4+ counts over time for patients within the outpatient monitoring system of the National AIDS Control Program in Tanzania. Each circle represents one patient. Blue value in bottom right of each panel showing the R^2^ for the correlation between these values, indicating that patients that start earlier do not gain more CD4+ cells, rather they reach greater asymptotic CD4+ counts because they start at greater initial counts.
